# Development of a Serological Assay for the Sea Lion (*Zalophus californianus*) Anellovirus, ZcAV

**DOI:** 10.1038/srep09637

**Published:** 2015-05-12

**Authors:** Elizabeth Fahsbender, Karyna Rosario, John P. Cannon, Frances Gulland, Larry J. Dishaw, Mya Breitbart

**Affiliations:** 1 University of South Florida, College of Marine Science, 140 7^th^ Avenue South, Saint Petersburg, FL 33701 USA; 2 University of South Florida, College of Medicine, Department of Pediatrics, 801 6^th^ Street South, Saint Petersburg, FL 33701 USA; 3 The Marine Mammal Center, 2000 Bunker Road, Sausalito, CA 94965 USA

## Abstract

New diseases in marine animals are emerging at an increasing rate, yet methodological limitations hinder characterization of viral infections. Viral metagenomics is an effective method for identifying novel viruses in diseased animals; however, determining virus pathogenesis remains a challenge. A novel anellovirus (*Zalophus californianus* anellovirus, ZcAV) was recently reported in the lungs of captive California sea lions involved in a mortality event. ZcAV was not detected by PCR in the blood of these animals, creating the inability to assess the prevalence of ZcAV in live sea lions. This study developed an enzyme-linked immunosorbent assay (ELISA) to detect antibodies to ZcAV in sea lion serum. To assess ZcAV prevalence, paired serum and lung samples (n = 96) from wild sea lions that stranded along the California coast were tested through ELISA and PCR, respectively. Over 50% of the samples tested positive for ZcAV by ELISA (34%), PCR (29%), or both (11%) assays. ZcAV is prevalent in stranded wild sea lion populations and results suggest that PCR assays alone may grossly underestimate ZcAV exposure. This ELISA provides a tool for testing live sea lions for ZcAV exposure and is valuable for subsequent studies evaluating the potential pathogenicity of this anellovirus.

The rate of emergence of new diseases in marine animals is increasing^1^,^2^,^3^,^4^, generating a need for surveillance of potential pathogens to protect marine mammals against epidemics. However, it remains difficult to characterize and diagnose viral infections because of methodological limitations^5^. Current detection methods such as degenerate PCR and pan-viral microarrays can detect close relatives of previously described viruses, but are limited for detecting novel viruses. Viral metagenomics (virus particle purification followed by shotgun sequencing) is an effective method for identifying viruses involved in mortality events in marine animals^6^,^7^,^8^,^9^, yet it remains difficult to establish a connection between a novel virus and disease due to limitations of culturing the viruses as well as difficulties in obtaining fresh diagnostic tissues from wild marine mammals.

Viral metagenomics performed on lung tissue of several necropsied captive California sea lions (*Zalophus californianus*) with signs of respiratory disease that were involved in a mortality event of unknown cause revealed the presence of a novel anellovirus, *Zalophus californianus* anellovirus (ZcAV)^7^. ZcAV was found by specific PCR in the lungs of all three of the sea lions that died in the mortality event, but it was not found in sea lions from the same zoo that died of unrelated causes. In addition, ZcAV was found to be actively replicating in lung tissue of a sea lion from the mortality event, further suggesting an association of ZcAV with the death of these animals. In addition to the captive sea lions, 11% of lung samples from wild sea lions stranded off the California coast tested positive for ZcAV by PCR, indicating that this anellovirus is present in wild populations.

The initial ZcAV discovery and prevalence studies have raised many questions about the potential pathogenicity of this virus. Anelloviruses have been extensively studied in humans, where these viruses can be highly prevalent (infecting up to 100% of the population)^10^,^11^,^12^,^13^, yet they have not been linked to human disease. Although anelloviruses have also been found in a wide range of mammals including non-human primates^14^, domestic animals^15^, Pacific harbor seals^9^, and Risso's dolphins (E.M. Fahsbender et al., unpublished data), anellovirus-associated pathology remains unknown. Initial evidence suggested that ZcAV may be linked to the mortality event of captive California sea lions; however, studies investigating the pathogenicity of this virus are difficult since ZcAV has only been detected by PCR in tissues of necropsied animals, and cannot be detected by PCR in the blood of infected individuals^7^. The inability to detect ZcAV in blood samples by PCR severely limits further testing for this virus, since obtaining lung biopsies from live sea lions is not possible. Hence, there is a critical need for an assay to detect ZcAV exposure in blood samples to investigate the epidemiology of this virus, understand its association with disease, and preemptively develop management strategies that can prevent the spread of this virus in captive and rehabilitation animals. To overcome these technical limitations of studying the role of ZcAV in disease, here we describe the development of an enzyme-linked immunosorbent assay (ELISA) for ZcAV, and demonstrate that sea lions mount an immune response against ZcAV.

Similar to other anelloviruses^16^, ZcAV contains a small (2140 nucleotide (nt)), negative sense, single-stranded DNA circular genome that encodes three major open reading frames (ORFs). Based on similarities to other anelloviruses, ORF 1 is believed to encode the capsid protein, although this has not been experimentally demonstrated for any anellovirus. For other anelloviruses, the ORF 1 gene product has been predicted to be antigenic due to the presence of major hydrophilic regions, and ORF 1 has been successfully used in seroprevalence studies in humans and pigs^11^,^17^,^18^,^19^,^20^. Here we developed an ELISA based on hydrophilic regions of the ORF 1 gene product of the ZcAV genome and demonstrated that this assay is capable of detecting anti-ZcAV antibodies in sea lion serum. This ELISA provides a tool for studying ZcAV epidemiology and identifying seroconversion upon symptom development in captive sea lions, enabling future research investigating the pathogenesis of ZcAV. Finally, the creation of this assay lays the groundwork for bridging the gap between genome discovery via viral metagenomics and assessing the epidemiology of novel viruses and their significance for wild populations.

## Methods

### Sample collection

Paired serum and lung samples were collected from 100 California sea lions involved in stranding events along the coast of northern California. All samples were collected by The Marine Mammal Center located in Sausalito, California in accordance with Marine Mammal Protection Act permit no 932-1905/MA-009526 to Gulland. Serum samples were collected during routine clinical examinations of live stranded sea lions as described by Bossart et al.^21^ and archived at −70°C. Lung samples were collected from the same animals upon necropsy, with animals stored at 4°C between time of death and necropsy (within 24 hours of death). All protocols were approved under the Marine Mammal Protection Act. Paired lung and serum samples were shipped to the University of South Florida and stored at −80°C for processing using PCR (for lung samples) and ELISA (for serum samples).

### PCR testing of sea lion lung samples

Lung samples were tested with a ZcAV-specific PCR assay. For this purpose, DNA was extracted from approximately 25 mg of sea lion lung tissue using the DNeasy Blood and Tissue Kit (Qiagen). DNA was amplified through rolling circle amplification (RCA) using the Illustra Templiphi Kit (GE Healthcare), which is known to enrich for small circular templates such as the ZcAV genome^22^,^23^. RCA products were then used as the DNA templates for the PCR assay using primers ZcAV1056F (5′-AGG CAC TCA CAT AAT CTA TTC AA-3′) and ZcAV1369R (5′-CCC AGG CAT TAC AGG CTT TA-3′) designed to target a 314 nt region from ORF 1 of ZcAV. The PCR [containing 1 µM of each primer, 200 µM of dNTPs, 1 U of Red *Taq* (Sigma-Aldrich), 1X Red *Taq* reaction buffer and 2.5 µl of DNA template in a 25 µl reaction] proceeded as follows: 95°C for 5 minutes, 45 cycles of [94°C for 1 minute, 54°C for 1 minute, and 72°C for 1 minute], followed by a final extension at 72°C for 10 minutes. PCR products of the correct size were verified by gel electrophoresis. Four samples were cloned using a TOPO TA Cloning Kit (Invitrogen) and commercially sequenced. Sequences were then compared against the GenBank database in order to verify the specificity of the assay. To determine assay sensitivity, a positive control was prepared as previously described. The PCR product was cleaned using the UltraClean PCR Clean-Up Kit (Mo Bio Laboratories) and quantified using a NanoDrop ND-1000 apparatus (NanoDrop Technologies). The number of targets per microliter was back-calculated and the positive control was serially diluted to determine the sensitivity of the assay, which was 10 targets.

### Optimization of ELISA parameters

An ELISA was developed to screen sea lion serum for antibodies to ZcAV. Since the virus was identified from a mixed community using metagenomics, there were no purified virus particles that could be used as an antigen for the ELISA. Therefore, the ELISA was developed based on regions predicted to be immunogenic based on hydrophilic sections found in the predicted protein sequence of ORF 1. Four peptides (on average, 25 amino acids in length) from different regions of ORF 1 were synthesized at 70% purity by Pacific Immunology. Each of these peptides was scrambled, synthesized and used as a control peptide. Sea lion sera were used as primary antibodies, while alkaline phosphatase (AP)-conjugates of protein A (Roche) and protein L (Thermo Scientific) were used as detection reagents. Protein A is a bacterially-derived molecule that binds to the Fc region of IgG of many mammalian species; in contrast, protein L, also derived from bacteria, binds to immunoglobulin light chains and thus has the ability to detect a wider range of serum antibodies, including IgG, IgM, IgA and IgE^24^,^25^,^26^. Similar reagents have been used previously in sea lion serological testing^26^. Checkerboard titrations were performed to find optimal concentrations of peptide, serum, and proteins. After initial screening, peptide 4T (amino acid sequence: GMENTPPKRVRFRQSDVLRKHKHRI), along with its scrambled control peptide 4C (amino acid sequence: QRLHPKKHIRSETKFVRVDRNPGMR) were chosen as primary targets due to their low background. Protein L was selected as the detection reagent since it is able to bind more classes of immunoglobulins than protein A.

### ELISA testing of sea lion serum samples

High binding 96-well microtiter plate wells were coated with a final concentration of 1 µg/ml peptide diluted in 0.5 M carbonate buffer (pH 9.6) (1.59 g of Na_2_CO_3_, 2.93 g of NaHCO_3_, 0.2 g of NaN_3_, and dH_2_O to 1 liter) and the plate was incubated overnight at 4°C. All wells were washed three times with a phosphate-buffered saline (PBS) solution containing 0.1% Tween-20 (PBS-tween) using an automated microtiter plate washer, and then blocked with 1% bovine serum albumin (BSA) diluted 1:5 in PBS-tween for 1 hour. Sea lion serum samples were diluted 1:50 in PBS-tween containing 1% BSA, added to each well in triplicates, and left to incubate overnight at 4°C. The plate was then washed and blocked as previously described before secondary reagent protein L was added in PBS-tween containing 1% BSA at a 1:10,000 dilution and left to incubate at 37°C for 1 hour. Each well was washed as previously described. ELISA Blue (SureBlue Reserve) was added and incubated at room temperature for 10–20 minutes. Plates were read on a PerkinElmer Enspire machine to measure optical density (OD) at 650 nm.

### Statistical Analysis

The statistical software package GraphPad Instat (GraphPad Software, Inc.) was used to perform an analysis of variance (ANOVA) on averages of the triplicate sea lion serum samples. Samples with p-values >0.05 were considered statistically significant. Samples were considered positive if serum incubated with the test peptide (4T) had a statistically significant higher OD value than the serum incubated with the control peptide (4C).

## Results

Development of the ELISA using serum samples from sea lions whose lung samples were PCR-positive for ZcAV demonstrated that sea lions mount an immune response to ZcAV infection. Paired serum and lung samples from 100 stranded California sea lions were then tested for the presence of ZcAV nucleic acids and anti-ZcAV antibodies through PCR and the newly developed ELISA, respectively. Four samples were removed from the data set due to high background with the ELISA. Of the 96 samples tested, 50 (52%) tested positive for ZcAV exposure (ELISA; serum samples; n = 33 [34%]) and/or DNA presence (PCR; lung samples; n = 28 [29%]) (Table 1). Eleven percent of the sea lions tested positive for ZcAV by both ELISA and PCR, and 48% of sea lions tested negative by both methods. Of the 28 sea lions with PCR-positive lung samples, 39% were also serum-positive by ELISA. However, 32% of the PCR-negative sea lions (n = 68) also tested positive for ZcAV exposure via ELISA. The Kappa value of 0.066, calculated using GraphPad, indicates poor correlation between the PCR and ELISA test results.

## Discussion

The ELISA developed in this study provides the first and only method to test for ZcAV exposure in live sea lions. Here, this assay was used in combination with PCR to assess the prevalence of ZcAV in the wild, stranded sea lion population along the northern coast of California. Protein-L-based detection of ZcAV-specific antibodies suggests the exposed sea lions are able to recognize and mount immune responses to the virus. These data, which are the first obtained for marine mammal anelloviruses, support prior studies showing that human anelloviruses are immunogenic, with both IgG and IgM class antibodies produced upon exposure to ORF 1^27^,^28^. The combined ELISA and PCR results demonstrate that ZcAV infections are prevalent and persistent in wild California sea lions.

The development of this ELISA was unconventional due to the many difficulties of working with marine mammals. Gaining access to 100 paired serum and lung samples from a protected species is both arduous and time consuming. Furthermore, the sea lion immune system is not frequently studied; therefore, there are no commercial antibodies for sea lions. In addition to the many ambiguities of the sea lion immune system, ZcAV is not a well-understood virus. ZcAV was discovered from a mixed community and attempts to isolate ZcAV virions in culture have been unsuccessful, limiting sensitivity testing of this ELISA. In addition, without more knowledge of the types of related anelloviruses found in marine mammals, the specificity of this assay cannot be determined.

Although the ELISA and PCR results obtained in this study were not correlated, the ZcAV ELISA provides complementary results to the PCR data, providing a greater scope to measure virus prevalence, as use of the ZcAV ELISA increased the detection of exposure to ZcAV. There are multiple explanations for the incongruences between the ELISA and PCR data. ELISA-positive, but PCR-negative samples (23%) are likely due to previous ZcAV infections in which the viral DNA is no longer present but the ELISA can detect the presence of (long-lived) antibodies, suggesting a prior exposure. Similar results have been seen for the human anellovirus Torque teno virus (TTV), where antibodies were detected despite the absence of TTV DNA^11^. In addition, if the ZcAV infection occurred in another sea lion organ instead of the lungs, we could expect a PCR-negative but ELISA-positive result. Conversely, sea lion samples may have tested positive by PCR but negative by ELISA (18%) if the sea lions were actively infected upon stranding and died before a detectable antibody response was mounted. Although the timing of the response of the sea lion immune system to ZcAV is unknown, development of the human antibody response to TTV is slow and known to take up to 21 weeks after virus inoculation^29^, although once the antibodies are produced they persist in serum for long periods^28^,^30^. Finally, slight sequence mutations or antigenic diversity of anelloviruses may also contribute to data incongruency. Anelloviruses are known to demonstrate a high degree of genetic variability^19^,^31^,^32^ which can pose problems for both DNA detection by PCR primers and antibody detection. For certain TTV genotypes, it has been shown that multiple serotypes may exist in circulation^20^. Future work should compare the diversity of anelloviruses that can be detected by the PCR and ELISA utilized in this study, as the discrepancy between the two assay types may reflect differences in the diversity of genotypes and serotypes recovered.

Through a combination of PCR- and ELISA-based testing, this study demonstrated that sea lions mount an immune response to the anellovirus ZcAV and revealed the high prevalence of ZcAV in a stranded wild sea lion population. The ELISA created here will enable future research on the epidemiology of ZcAV in live sea lions and allow further study of ZcAV pathogenesis by measuring seroconversion in infected captive sea lions. Finally, this assay has direct implications for protecting sea lion health since it is the only method available for the detection of ZcAV in live sea lions. The ZcAV ELISA enables the screening of rehabilitation animals before admitting them into facilities with healthy animals, which can prevent the spread of this virus to other captive sea lions and provide a valuable management step if further work links ZcAV with disease.

## Figures and Tables

**Table 1:** Prevalence of ZcAV in paired serum and lung samples from a stranded wild population of California sea lions (*Zalophus californianus*; n = 96) assessed by ELISA and PCR, respectively

	Serum ELISA positive n = (%)	Serum ELISA negative n = (%)	PCR totals n = (%)
Lung PCR positive n = (%)	11 (11%)	17 (18%)	28 (29%)
Lung PCR negative n = (%)	22 (23%)	46 (48%)	68 (71%)
ELISA totals n = (%)	33 (34%)	63 (66%)	
